# Why are anopheline mosquitoes not present in the Seychelles?

**DOI:** 10.1186/1475-2875-10-31

**Published:** 2011-02-08

**Authors:** Vincent Robert, Gérard Rocamora, Simon Julienne, Steven M Goodman

**Affiliations:** 1 MIVEGEC Maladies infectieuses et Vecteurs: Ecologie, Génétique, Evolution et Contrôle (IRD 224, CNRS 5290, Université Montpellier 1, Université Montpellier 2), Centre IRD France-Sud, BP 64501, 911 Avenue Agropolis, 34394 Montpellier cedex 5, France; 2 Island Conservation Society, PO Box 775, Pointe Larue, Mahé, Seychelles; 3 Ministry of Health, Victoria, Mahé, Seychelles; 4 Field Museum of Natural History, 1400 South Lake Shore Drive, Chicago, Illinois 60605, USA and Vahatra, BP 3972, Antananarivo 101, Madagascar

## Abstract

**Background:**

Species of anopheline mosquitoes are largely distributed over emerged lands around the world and, within the tropics, few areas are without these insects, which are vectors of malaria parasites. Among the exceptions is the Seychelles archipelago in the western Indian Ocean. However, in the Aldabra island group, located in the extreme western portion of the archipelago, *Anopheles gambiae s.l. *was introduced, leading to massive proliferation and then elimination, with the most recent autochthonous malaria cases recorded in 1931.

**Methods:**

In order to re-examine the absence of anopheline mosquitoes in the Seychelles, an entomological field survey was conducted in December 2008 at 17 sites on four granitic islands, including Mahé and Praslin, and ten sites on coralline atolls in the extreme west, including Aldabra.

**Results:**

No evidence of larval or adult anophelines was found at the surveyed sites, which supports their absence in the Seychelles.

**Conclusions:**

In the granitic islands of the Seychelles, the climate is favourable for anophelines. However, these islands are protected by their remoteness and prevailing seasonal winds. In addition, stagnant freshwater, required in anopheline larval development, is relatively uncommon on the granitic islands because of the steep slopes. In the southwestern atolls (Aldabra and Providence-Farquhar groups), the presence of a long dry season of up to nine months and the total absence of permanent natural freshwater prevents the breeding of anophelines and their successful colonization. The Seychelles does not have any native land mammals and like in other parts of the world (Antarctica, Iceland, New Caledonia, Central Pacific islands) their absence is associated with the lack of anophelines. This suggests an obligatory relationship for anophelines to feed on terrestrial mammals, without alternative for blood-feeding sources, such as bats, birds and reptiles.

## Background

*Plasmodium *parasites are transmitted by mosquitoes (family Culicidae). Malaria parasites of mammals, including humans, are exclusively transmitted by mosquitoes belonging to the genus *Anopheles*. These anophelines are observed virtually worldwide, although the distribution of a given *Anopheles *species varies from being highly localized to sub-continental. For example, on the one hand *Anopheles bwambae *occurs exclusively within a 10 km radius of geothermal springs in Bwamba, Uganda, and, on the other hand, *Anopheles messeae *is found in most of Eurasia from Ireland to Central Siberia [1,2].

The examples of areas without anophelines are limited and include Antarctica, Iceland, and a heterogeneous set of tropical islands, including the Seychelles, New Caledonia and those in the Central Pacific. In the first two zones, there are simply no mosquitoes, and in the third category there are no anophelines (sub-family Anophelinae), although culicine mosquitoes (sub-family Culicinae) abound. In this list of islands without anophelines, the case of the Seychelles is puzzling for at least two reasons: (i) the Seychelles is located in the southwestern Indian Ocean where most existing islands, if not all, present autochthonous or introduced anophelines (African east coast, Madagascar, the Comoros archipelago, La Réunion, Mauritius); (ii) subequatorial islands in the Seychelles have climatic conditions suitable for anophelines. Consequently, it is difficult to explain the absence of anophelines in the Seychelles [3,4].

### An overview of the Seychelles

**Figure 1:**
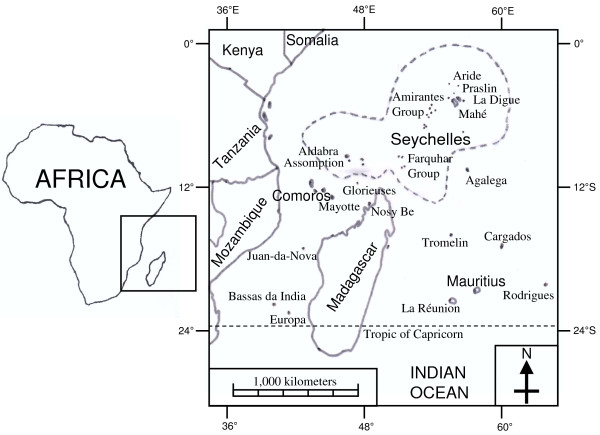
**Map of the Seychelles islands**. The Seychelles islands and the main neighbouring emerged lands. Dashed line represents the limit of the Seychelles' Exclusive Economic Zone.

The Seychelles is an island state. The archipelago comprises 84,000 inhabitants (2009 census), making it the smallest population of any African state. The total emerged surface area is 455 km^2^, composed of about 115 main islands located 4° to 10° south of the equator. The majority of islands are without any permanent residents. Islands are grouped in several distant archipelagos, which comprise a zone of about 1,400,000 km^2 ^(Figure 1). The largest island of Mahé (surface area of 155 km^2^) hosts the capital city, Victoria (26,000 inhabitants), the international airport and the principal harbour. The Seychelles is listed amongst the biodiversity hotspots of the world [5], with high levels of endemism and law protects almost 50% of the archipelago land surface as nature reserves or national parks. However, there are no native mammals in the Seychelles, with the exception of bats [6,7]. As in most areas of the world, where humans have established themselves, domestic or commensal mammals, such as cattle, dogs, cats, rats and mice, have been introduced.

**Figure 2:**
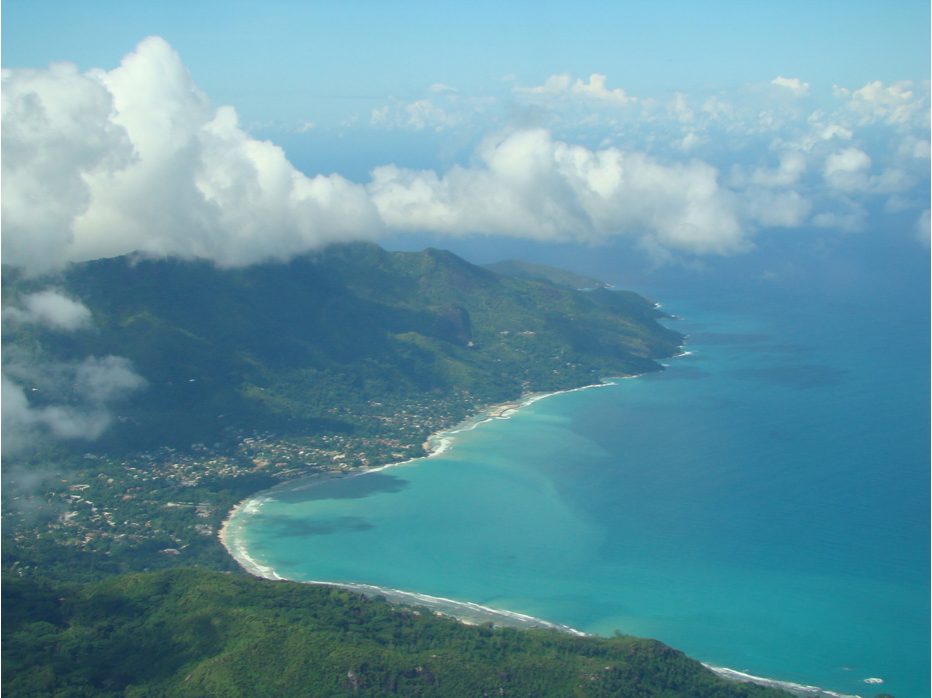
**Photo of Mahé, the main granitic island in the Seychelles**. Note the important elevational gradient and steep slopes of most of the island. (Photo V. Robert/IRD).

**Figure 3:**
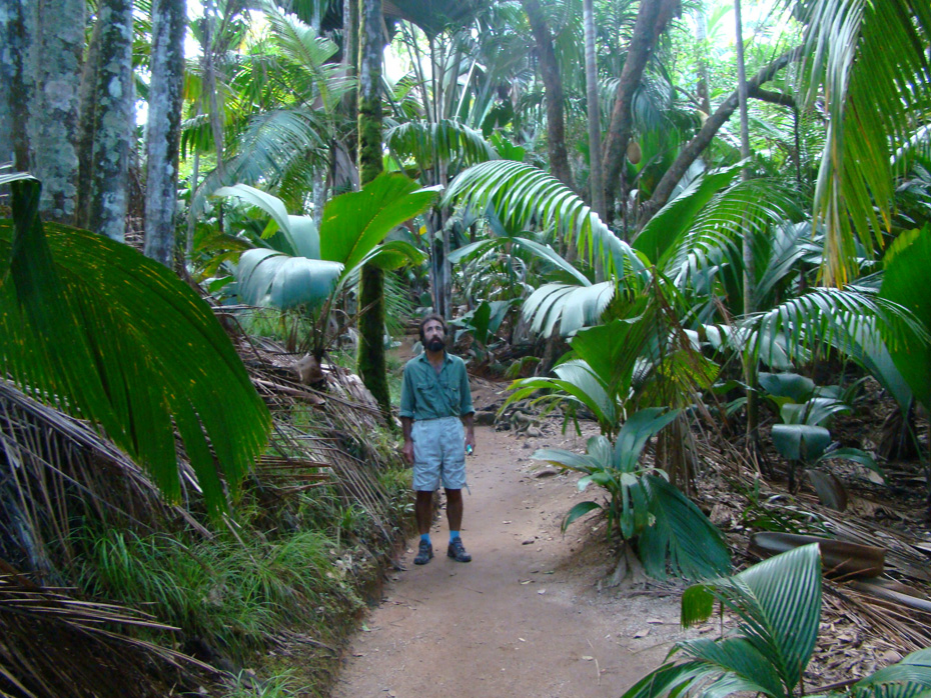
**Photo of Vallée de Mai, Praslin Island**. Note the flora, composed largely of palm trees. (Photo V. Robert/IRD).

The archipelago can be divided into two distinct types of islands, based on their geological origin, granitic and coralline. The granitic islands are located in the northeastern part of the Seychelles, about 1,000 km northeast of Madagascar and 1,500 km east of Kenya, and comprise three large islands (Mahé, Praslin and La Digue) on which 99% of the human population lives. The granitic portions of the Seychelles are the only mid-oceanic granitic islands in the world and are remnants of the break-up of ancient Gondwana supercontinent associated with the splitting of Indo-Madagascar [8,9]. These granitic islands have been separated from other emerged land for c.75 million years. Given their elevation, in geological time these islands were never totally submerged associated with rising sea-levels, although in some cases modern separate landmasses, which rest on a submarine microcontinental plateau, would have been connected during periods of lower sea-level (Figure 2). Mahé has the highest peak in the archipelago, Morne Seychellois at 930 m. This mountain mass holds extensive humid tropical forests dominated by exotic plants, but with relict largely primary forests above 600 m. The Vallée de Mai on Praslin has native forest, composed by a variety of endemic palm trees (Figure 3).

The climate on the granitic islands is very humid (≥80%) all year round and on Mahé average temperatures vary little throughout the year, from 24 to 30°C at sea-level, and annual rainfall ranges from less than 1.9 m in the south, 2.7 m at Victoria and over 3.6 m on the mountain slopes. During the coolest months, July and August, the average temperature drops a few degrees. The hot months, December to April, have maximum temperatures in March and April (up to 34°C). The southeast trade winds blow regularly from May to November. Most of the archipelago lies outside the cyclone belt, so very strong winds are rare.

**Figure 4:**
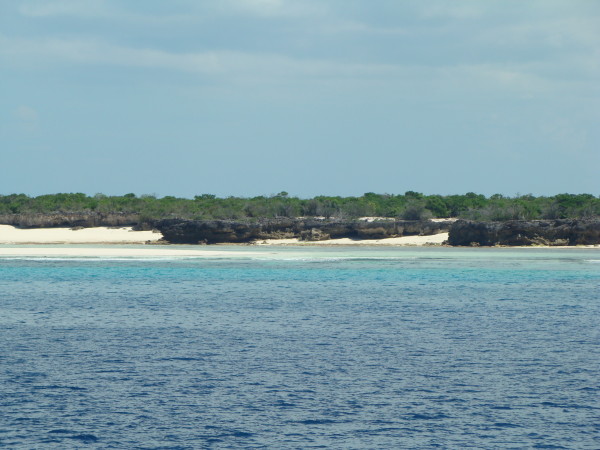
**Photo of Aldabra Atoll, Island of Grande Terre**. Note the low elevation of the coralline island rising just above sea level. The local peak is at 18 m a.s.l. (a sand dune) and the average altitude is between 2 and 3 m. (Photo V. Robert/IRD).

Coralline islands are situated beyond the Seychelles Plateau and grouped into several archipelagos, the Amirantes group, the Farquhar group and the Aldabra group. They present calcareous substratum on volcanic bedrock and have a maximum elevation above sea-level of a few meters. Consequently, they were totally submerged during marine transgressions, periods that would have eliminated the terrestrial biota. For instance, in recent geological history, Aldabra was completely underwater on at least two occasions, with the most recent complete submersion occurring c.125,000 years ago [10-12]. Aldabra is a huge atoll (155 km^2 ^of emerged land) of 34 km long, 14.5 km wide; it consists in raised coral of up to 8 m above sea level (Figure 4), and comprises 4 main islands enclosing a central lagoon of 224 km^2 ^[13]. The greatest colony in the world of giant tortoises (*Testudo gigantea) *is found at Aldabra, with up to 150,000 individuals. The avifauna is very diversified for both landbirds and seabirds, the latter being most abundant in the mangroves and islets around the lagoon [14].

The climate of the coralline islands is rather different from the granitic islands. For instance, on Aldabra, from April to October, the trade winds blow from the southeast and carry relatively dry and cool air, with minimal temperatures of 22°C in August. From November to March, the monsoon coming from northwest brings higher humidity and temperatures with maximums up to 32°C in December. Annual mean temperature is 27°C. Annual mean recorded rainfall at Aldabra-Picard is 1,100 mm, with ranges varying from 349 to 1,467 mm during the period 1949-1978.

The islands of Aldabra and Mahé are 1,150 km distant from one another, but the southwestern islands are much closer to neighbouring foreign countries, with Farquhar at c. 290 km from Madagascar and Aldabra at c. 420 km equidistant from the Comoros and Madagascar. Assomption Island belongs to the Aldabra group, at some 40 km south of the Aldabra Atoll, and is nearly 7 km long and 2.5 km wide.

### Current knowledge on mosquitoes and main associated diseases in the SW Indian Ocean and in the Seychelles

In the southwestern Indian Ocean (SWIO), all the culicidian vectors (but not all mosquitoes) are of exogenous origin. This includes Madagascar where the rich endemic culicidian fauna contains 13 species of anophelines, not a single one being a known vector of malaria parasites (Table 1). In this SWIO area, there is no endemism at the generic level, and all endemic species belong to the genera *Culex*, *Aedes *and *Anopheles*, which have African or Malagasy affinities [15].

**Table 1:** Number of anopheline species per country

Area	Nb of anopheline species
Afrotropical region	142
Somalia	20
Kenya	41
Tanzania	48
Mozambique	29
Comoros	8
Madagascar	26
La Réunion	3
Mauritius	5
Seychelles	0

Number of anopheline species per geographical area, including countries bordering the southwestern Indian Ocean [Source 61]

*Culex quinquefasciatus*, a pan-tropical mosquito, is present on all the SWIO islands. It can easily reproduce in the water supply of ships, permitting its introduction by early sailors who crossed the Indian Ocean. Contrarily to continental Africa and on any islands of the SWIO, *Cx. quinquefasciatus *is considered as the unique potential vector of the lymphatic filariosis *Wuchereria bancrofti *in the Seychelles, although its transmission capacity is poor [4] and where a dramatic decrease of this transmission has been noted in recent years (S. Julienne, unpublished data).

*Aedes aegypti *and *Aedes albopictus *are also mosquitoes that can be easily transported. They reproduce in small amounts of water. Their eggs are resistant to dehydration within receptacles where they are laid and, when re-immersed in water, they successfully hatch. At present, *Ae. aegypti *occupies all the islands of the SWIO, except Mauritius where it was eradicated as a result of a DDT spraying campaign between 1949 and 1951 to control malaria. On Madagascar, it occupies only the western coast. *Aedes albopictus *is present along the eastern Malagasy coast and on Mauritius. Recently it has been observed for the first time on Grande-Glorieuse (distant of only 135 km from Astove, in the Aldabra group) and Juan-da-Nova [16]. On La Réunion and the granitic islands of the Seychelles, both *Ae. albopictus *and *Ae. aegypti *occur, as in many portions of the world with the former being more common [17-20]. In the coralline portions of the Seychelles, only *Ae. aegypti *is documented. The Seychelles experienced epidemics of dengue II from December 1976 to April 1977 [21,22] and of Chikungunya from March 2005 to late 2007 [23] with *Ae. albopictus *as the main presumed vector of these diseases.

The case of anophelines is different. Over relatively short distances (up to a few hundred kilometres), adult mosquitoes can be transported by wind. However, most introductions of anopheline mosquitoes over long distances are thought to have occurred via human-aided means, especially accidental transportation on ships and aircrafts. *Anopheles *species that are efficient vectors of human malaria generally do not produce desiccation-resistant eggs, are not autogenous, breed in natural habitats, are not known to undergo diapause and do not reproduce on boats. For all these reasons, they have certain limitations, as compared to *Aedes*, for transportation across large distances. *Anopheles *can probably survive no more than ten days without laying eggs, which excludes long distance transport by boat. Indeed, members of this genus invaded Mauritius in 1865, after the commencement of steamer line from Tamatave (Madagascar) to Port Louis, which reduced the duration of the journey of one month to a few days (Table 2). On the other hand, it is unclear how and when anophelines became established on Madagascar and the Comoros. They may have been introduced by early sailors but the possibility of wind dispersion cannot be eliminated; indeed, the natural colonisation by *Anopheles arabiensis *of La Réunion from Mauritius (about 200 km away) associated with a cyclone prior to the first malaria outbreak on La Réunion in 1868 is a valuable clue [24,25]. At present, *Anopheles gambiae s.s*. is known from the Comoros and the eastern coast of Madagascar [26]. *Anopheles arabiensis *occupies the totality of Madagascar, Mauritius, La Réunion and Grande Glorieuse [27,28]. On Madagascar and the Comoros, except on Grande Comore, *Anopheles funestus *breeds in rice fields. This species was introduced to Mauritius and perhaps La Réunion, but disappeared during DDT treatments. The granitic islands of the Seychelles, the remote Rodrigues Island and the Chagos archipelago have no known anophelines.

**Table 2:** Well-documented introductions of anopheline species in the southwestern Indian Ocean

Date	Location	Anopheline	Main reference
1865	Mauritius	*An. gambiae s.l.*	[24]
1867	La Réunion	*An. arabiensis*	[24]
Before 1900	Mauritius	*An. coustani*	[62]
Before 1900	Mauritius	*An. maculipalpis*	[62]
Before 1902	La Réunion	*An. coustani*	[63]
1908	Aldabra (Seychelles)	*An. gambiae s.l.*	[30]
1930	Aldabra and Assomption (Seychelles)	*An. gambiae s.l.*	[30]
Before 1932	Mauritius	*An. funestus*	[24]
1949	La Réunion	*An. funestus*	[24]
Before 1975	Mauritius	*An. arabiensis*	[27]
1975	Assomption (Seychelles)	*An. gambiae s.l.*	[32]
2002	Grande-Glorieuse (France)	*An. arabiensis*	[28]

Finally, the culicidian fauna in the Seychelles contains 17 species in four genera (*Culex*, *Aedes*, *Mansonia *and *Uranotaenia*). Amongst these are two invasive mosquito species, *C. tritaeniorhynhus *and *C. fuscocephallus*, introduced to Mahé between 1969 and 1995 [29].

### The case of Aldabra

In the Aldabra group (composed of Aldabra, Assomption, Cosmoledo and Astove), the history of the presence of *Anopheles *and *Plasmodium *is complicated. Up until the beginning of the 20^th ^century, the island group had been unambiguously considered as safe with regards to malaria transmission. In March 1908, the first malaria outbreak of benign tertian type occurred on the Aldabra Atoll. The disease was confined to the settlement on Picard Island and started 11 days after the arrival of a boat from Nosy-Be (Madagascar) and with Malagasy passengers suffering from malaria. The number of infected humans gradually increased with a peak in July-August of that same year, but a progressive diminution in severity was noticed, the earlier cases being the more severe. The last incident was observed at the end of September 1908. In total, 98 cases were recorded, all treated by quinine [30]. Even though no specific research was conducted on mosquito larvae, by mid-September, the oiling of all water pools and puddles was performed on the atoll [31]. On the basis of fieldwork conducted by several eminent entomologists from October 1908 to January 1909 [JCF Fryer, 1910 in 30], no evidence of adult or larval *Anopheles *was detected.

After a lapse of two decades, a second malaria outbreak, this time due to *Plasmodium falciparum *imported via workers who were previously on Mauritius and Juan-da-Nova, occurred with a first phase on Assomption (June to October 1930) and a second phase on Aldabra (October 1930 to January 1931). It is an established fact that this outbreak was transmitted by a species of the *Anopheles gambiae *complex (named *costalis *at that time). During the epidemic, huge densities of this mosquito were observed at adult and larval stages on Picard Island, but not documented on other islands of the atoll. Vector control targeting larvae was performed on 31 January 1931 using 'Paris green oil' (an insecticide routinely used at that time), poured into 966 pools of water in the neighbourhood of the Picard Island settlement, among which 281 had anopheline larvae. Anophelines were not documented on Assomption from the end of 1930, but still remained somewhat numerous on Picard, despite the insecticide treatment, until April 1931 [30]. These malaria cases were the last autochthonous ones recorded in the Seychelles.

These introductions of anophelines in the Aldabra group were apparently not followed by successful continuation of local populations over the following dry season. It has been questioned if *An. gambiae s.l. *can survive on Aldabra during the extended dry season at sufficient densities to cause subsequent malaria outbreaks, perhaps feeding on numerous wild goats. Presumably, the lack of freshwater pools on the island during the dry season results in the crash and extirpation of local populations. Hence, each malaria outbreak is associated with an independent colonization event of the vector mosquito.

The last point to mention is the observation of *An. gambiae s.l. *on Assomption Island in 1975 [32]. This record must be interpreted with caution because complementary field surveys during the same period did not find additional evidence of anophelines on the island. The 1975 record of this mosquito on the island may have been due to materials that were previously used for training courses in continental Africa, resulting in accidental pipette contamination (S. Julienne, unpublished data).

Trying to explain why anopheline mosquitoes are not present in the Seychelles is difficult to answer. As a prerequisite, in order to provide insight into the dynamics of mosquito species richness and colonization/extinction patterns in the Seychelles, particularly for anophelines, a field survey of several different granitic and coralline islands was conducted, the results of which are presented here.

## Methods

A field survey was conducted in the Seychelles from 29 November to 18 December 2008, during the rainy season, a period *a priori *favourable to detect anophelines, if present. The islands chosen for survey were based on the following rationale. Four granitic islands were selected, including the three most populated (Mahé, Praslin, La Digue) and a small granitic island (Aride), the latter with only a handful of permanent human residents and over a million birds. Four coralline islands belonging to the Aldabra group were selected (Picard, Malabar and Grande Terre within the Aldabra Atoll, plus neighbouring Assomption). Emphasis was placed on these latter islands based on knowledge of previous anophelines introductions during the 20^th ^century. The list of visited islands and precise details of sampled sites are presented in Table 3.

**Table 3:** Principal results of the 2008 entomological survey for adult Culicinae mosquitoes in the Seychelles

Date	Island type	Island and locations for CDC light-trap (and Nb of night-trap)	Nb of adult Culicinae	Locations for larval sampling effort
Nov 29	Granitic	**Mahé **(145 km^2^)		Marre aux cochons
Nov 30		Victoria (2)	3	Tea plantation, Morne Blanc, Copolia
		Copolia (6)	4	Tea plantation
		**Aride **(0.7 km^2^)		
Dec 2		Plateau and summit (6)	5	Plateau, summit
		**Praslin **(38 km^2^)		
Dec 3		Anse Kerlan (6)	191	Grande Anse
Dec 4		Vallée de Mai (3), Fond B'Offay (1), Grande Anse (1)	41+1+30	Vallée de Mai, Baie Sainte-Anne, Marie Jeanne, Côte d'Or, Anse Boudin
		**La Digue **(10 km^2^)		
Dec 5		La Passe (4), La Veuve Reserve (2)	23+48	La Passe, La Veuve Reserve
Dec 6		L'Union (4), Pointe Source d'Argent (2)	57+11	L'Union, Pointe Source d'Argent
		**Mahé**		
Dec 7		Petit Paris (2)	5	
Dec 8		Anse Intendance (2), Baie Police (2)	46+130	
Dec 9		Baie Lazare (2), Port Launay (2)	15+2	

Total granitic islands		4 islands; 17 sites (48)	612 mosq	16 sites

	Coralline	**Aldabra - Picard **(9 km^2^)		
Dec 12		Research station (5)	19	Research station
		**Aldabra - Malabar **(27 km^2^)		
Dec 13		Middle camp (4)	130	Middle camp
		**Aldabra - Grande Terre **(116 km^2^)		
Dec 14		Takamaka grove (4)	1,154	Takamaka
Dec 15		Cinq Cases (1+3)	8,750*+1,800	Cinq Cases
Dec 16		Anse Maïs (3)	307	Anse Maïs
		**Aldabra - Picard**		
Dec 17		Jelly Fish Pound (1), Bassin Livine (1), Passe Femme (2)	10+0+22	Jelly Fish Pound, Bassin Livine
		**Assomption **(11 km^2^)		
Dec 18		Port (1), airport (3)	2,770*+104	Port; airport

Total coralline islands		4 islands; 10 sites (28)	15,066 mosq	9 sites

Grand total		8 islands; 27 sites (76)	15,678 mosq	25 sites

*estimate

**Figure 5:**
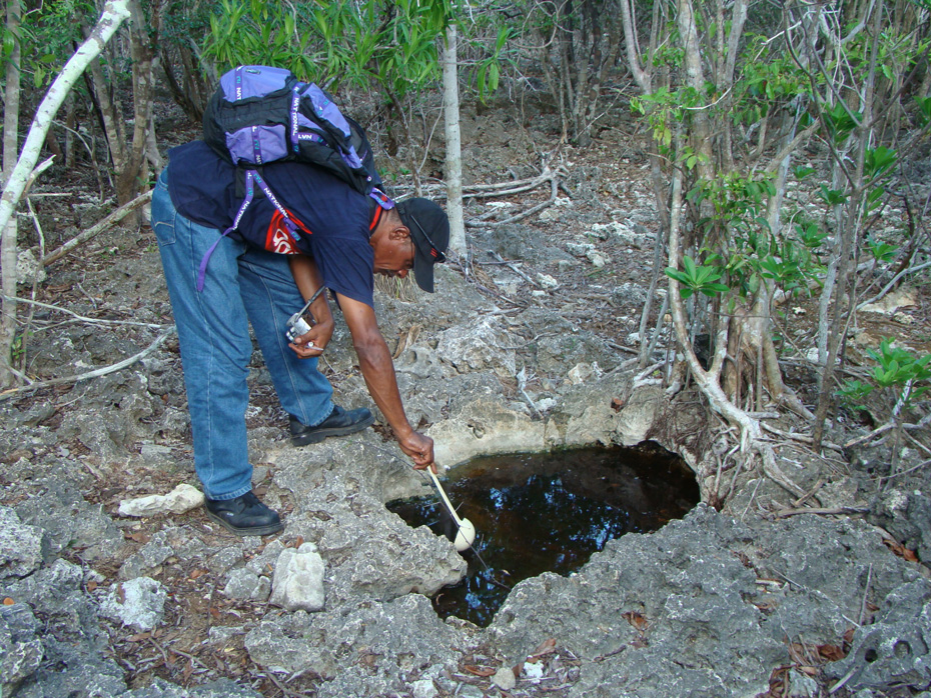
**Photo of mosquito larvae collection, Aldabra Atoll, Picard Island**. Note the larval breeding places are mainly natural and composed of coral rock pools inundated by rainwater. (Photo V.Robert/IRD).

Entomological survey methods included: (i) Immature stages (larvae and pupae) - detailed examination for immature stages was performed at natural and human-made water collection sites, including marshes, tree holes, crab holes in mangroves, containers and reservoirs (Figure 5); (ii) Adult mosquitoes - using CDC Miniature Light Trap 6 volts (BioQuip™) from dusk to dawn, with a 4-watt incandescent light and a 4-watt black light tube (UV light ca. 320-420 nm). CDC traps were placed outdoors and used without olfactory attractant such as CO_2 _or next to human/animal bait. Systematic collection of mosquitoes on humans was not conducted, although alighting mosquitoes on members of the field team were collected. Netting of insects resting on vegetation was conducted only on Mahé, with few captures and these are not reported on herein.

## Results

### Granitic islands (Mahé, Praslin, La Digue and Aride)

The number of sites with collection of adult and immature mosquitoes was 17 and 16, respectively. Forty-eight trap-nights were accrued, and total numbers of mosquitoes collected by traps and landing on humans were 612 and 16, respectively. In the context of pre-imaginal surveys, efforts concentrated on possible unknown endemic species of anopheline in the Mahé mountains, particularly larval stages living in small streams. This type of ecological setting on Madagascar is often occupied by endemic anophelines [33]. No evidence of anophelines was found on the granitic islands during these surveys (Table 3).

### Coralline islands (Picard, Malabar, Grande Terre and Assomption)

The number of sites with collection of adult and immature mosquitoes was 10 and 9, respectively. Twenty-eight trap-nights were accrued, and total numbers of mosquitoes collected by traps and landing on humans were 15,066 and 23, respectively. No anopheline was obtained on coralline islands.

## Discussion

Despite the effort of prominent entomologists [3,17,34,35] and the November to December 2009 field surveys, introduced and native anophelines have not been documented on the granitic islands. Malaria vectors are believed to never have existed on the granitic islands of the Seychelles and no recent evidence has been found of them on the coralline islands. This has important bearing for public health and the sector of the local economy depending on tourism.

### Hypothesis to explain the absence of anophelines in the Seychelles

The absence of anophelines in the Seychelles is surprising given that the climate is favourable for these mosquitoes at least on the granitic islands and to a lesser extent in the Amirantes archipelago. Two hypotheses can explain this absence of anophelines: (i) no natural colonization or introduction, and (ii) introduction did not result in the establishment of a viable breeding population.

Health control procedures currently in place in the archipelago at the few main points of entry have certainly reduced the possibility of successful introduction of mosquitoes, although this is difficult to quantify. For example, it is compulsory for all in-coming flights to the Seychelles to have the cabin sprayed with insecticide. Active control procedures by both customs and public health officers are in place in Victoria (Mahé) at the only international airport in the country and the port. Further, all boats arriving from outside of the country and their cargos are systematically inspected and a health certificate subsequently delivered. In certain cases, fumigation of containers and the complete vessel may be required. A programme aiming at developing biosecurity measures, including training and improvement of enforcement, is currently being implemented as part of a GEF Biosecurity Project. The Seychelles are an important tourist destination with over 150,000 individuals arriving per year (estimation for 2007); three quarters of them being Europeans [23]. With the exclusion of the Victoria port, most of the harbours in the country are small, mainly devoted to local fisheries and national commerce. The majority of international flights provide connections to Europe and the Middle East, with few connections to continental Africa and Asia. The dozen or so destinations served by Air Seychelles, the national company and main passenger carrier, includes countries without endemic malaria (Paris, Moscow, Singapore, Johannesburg, etc.), although flights to India (Mumbai, Chennai) are planned to commence in the near future. There has been no report of 'airport malaria'. It is hard to certify that the preventive measures installed by health authorities at the international airport and port are completely efficient, but it is reasonable to maintain these measures. The number of cruise ships coming directly to the Seychelles from Madagascar or Mayotte, both islands with endemic malaria, has been increasing over the last 15 years, at least until the recent decline due to piracy, and a procedure of fumigation of these vessels comparable to the system used for aircrafts may be advisable, especially when the vessels stay at quay.

Several authors have previously alerted to the dangers that Mahé and the other granitic islands harbour introduced anophelines [3,17,34,36]. From an ecological point of view, this seems perfectly possible in the few marshy zones present on these islands.

Dispersal of mosquitoes can occur either naturally (by active flight or passive dispersal) or mediated by human activities (transportation by land, air or water). The possibility of natural dispersion by wind currents exists from continental Africa, Comoros and Madagascar, especially on the coralline southwestern islands. As previously noted, this mean of dispersal had been suspected for *An. gambiae *on Comoros and Madagascar and for *An. arabiensis *on La Réunion. The African vector *An. arabiensis *was introduced in Natal, Brazil, in 1930. Larvae or adult mosquitoes are believed to have travelled by air or more probably by steamer ship from Dakar, Senegal. This introduction had a major importance as a malaria vector with higher vectorial capacity than the autochthonous anopheline species, causing malaria outbreaks in 1930-1931 and a devastating epidemic in 1938-1939 across much of northeastern Brazil, especially along river courses [37]. This is only one example, among many, of important mosquito invasions that have taken place across the globe [see [38,39]].

Once an introduction has taken place, several different factors come into play for successful establishment of viable breeding populations, such as adaptation to the new environment and ability to cope with predators and competitors. For anophelines, several undocumented introduction events might have occurred on the granitic islands of the Seychelles, as has been evidenced in the Aldabra group. It is important to note that in the latter case these events have not lead to successful colonization with permanent breeding populations. Excluding the case of chemical treatment, unfavourable environmental conditions during the long dry season is a critical aspect that does not allow anophelines to complete properly the breeding cycle. This in turn demonstrates certain constraints for these introduced animals to adapt to notably different ecological conditions. A case in point is that an *An. arabiensis *population was found in March 2002 on Grande Glorieuse [28], but was absent by April 2008 [20].

### The question of endemic anophelines in the Seychelles (local speciation)

Beside the aspect of the introduction and subsequent establishment of anophelines in the Seychelles, remains the question of the absence of native anophelines. Given that the granitic islands have not been submerged since deep in geological time, it is biogeographically plausible for endemic anophelines to exist in this portion of the archipelago. Many of the small mountain streams, for example on Mahé, show climatological and topographical parallels to places on Madagascar with endemic anophelines; such sites are ideal for the development of the larval stages of these mosquitoes. While seemingly anomalous, no endemic anopheline has been documented in the Seychelles [see Results and [34]]. Endemic species of mosquitoes exist in the Seychelles, belonging to the genera *Culex*, *Aedes *and *Uranotaenia *[35].

Does the present day biota on the granitic islands represent the continental species that remained on the islands after continental drifting? Conversely, did a previous radiation of anophelines occur on these islands, followed by complete extinction? These questions remain open. But it is noteworthy that the divergence time for the major culicid lineages (Anophelinae vs. Culicinae) date to the early Cretaceous, 217 million years ago (CI: 229.50 - 192.19) [40], at a time much prior to the breakup of the ancient Gondwana supercontinent and the subsequent splitting of Indo-Madagascar and the deposition of the granitic islands of the Seychelles, some 75 million years ago [8,9].

On the Aldabra Atoll, insect diversity is estimated to exceed 1,000 species, of which a minimum of 23% are endemic (38% in the Aldabra group), and mainly derived from Malagasy lineages [41,42]. Given the complete submersion of the atolls by rising sea-levels over the past 125,000 years, this level of endemism amongst invertebrates is considerable, and illustrates the rapid differentiation and speciation processes taking place at Aldabra [e.g., [43,44]].

### Biogeography of zones without anopheline mosquitoes

In order to understand why no native anophelines occur in the Seychelles, it is worthwhile to examine other areas of the world also lacking these mosquitoes, which are notably limited to: Antarctica, Iceland, and a heterogeneous set of tropical islands, namely the Seychelles, New Caledonia and islands in the Central Pacific. In the two first areas, mosquitoes are completely lacking and in the third area, there are no Anophelinae although the Culicinae are present.

The absence of anophelines on Antarctica is easy to explain. Antarctica is the coldest place on Earth and due to the harsh climatic conditions, insects are scarce and less than 100 species of insects have been recorded [45]. Most of these are ectoparasites, like lice, which live in the feathers of birds and the fur of seals. Three species of chironomid midges (Diptera) and some springtails (Collembola) are the only free-living insects known from Antarctica. As a corollary to this point, there are no native terrestrial mammals on Antarctica.

There is also no mosquito known from Iceland, although these insects are common in portions of the world at the same latitude, such as northern Canada, Greenland and Scandinavia. There are two species of Culicinae on Greenland (*Aedes nigripes *and *Aedes impiger*), one on Svalbard and the Jan Mayen islands (*Ae. nigripes*), and 28 in Norway (including *Anopheles claviger*, *An. messeae *and *Ae. nigripes*). The reason why mosquitoes, especially *Ae. nigripes*, have not colonized Iceland may be due to variable climatic conditions on the island, with sudden temperature increases in the middle of winter that disturb hibernating pupal stage beneath ice, activating hatching of eggs into adult mosquitoes as soon as the ice melts. Adult mosquitoes suffer considerable mortality with subsequent drops in temperature [46]. There are also no mosquitoes on the Faroe and Shetland Islands where the climate is notably milder during the winter (mean temperature 3.5°C on the Faroes) than on Iceland [47-49]. These two islands lie in the path of strong northeast winds that are common throughout the year. With the exception of the arctic fox on Iceland, no species of native terrestrial mammal is known from these islands.

In New Caledonia, located in the western Pacific, about 20 species of mosquitoes have been recorded in the genera *Culex*, *Aedes*, *Mansonia *and *Tripteroides*, but anophelines are unknown [50]. This observation is noteworthy, as New Caledonia is peripheral to the distribution of the major malaria vector *Anopheles farauti *(Australia, Papua-New Guinea and Vanuatu) [2]. The absence of *An. farauti *on New Caledonia may be related with the island being just south of a line drawn between the two southern most localities, Australia and Vanuatu, in which *An. farauti *has been collected. The difference in latitude implies colder temperatures (average temperature at sea level in Vanuatu ranges between 24° and 26°C versus 22° and 24°C on New Caledonia). In addition, the unique geological nature of soils on the main island of New Caledonia, mostly ferralitic or ferritic soils overlaying ultrabasic rock, might be toxic for mosquitoes at larval stages and, therefore, helps to explain the local absence of anopheline mosquitoes. However, Laird [51] mentioned that *An. farauti *and *Anopheles sundaicus *(a malaria vector in Indonesia and in the Malaysian Peninsula) may find suitable larval breeding places on New Caledonia.

The islands in the central Pacific with few or no native mosquitoes and a high volume of commercial or military air and maritime traffic are particularly vulnerable to mosquito invasions [38]. Having said this, no anopheline is known from these islands. In Polynesia, 43 species of mosquitoes are recorded, belonging to 7 genera [52], but the genus *Anopheles *is absent. In the Hawaiian archipelago, mosquitoes were unknown until 1826, when a whaling ship introduced *Cx. quinquefasciatus*. In 2004, *Aedes japonicus japonicus *was found to be established and widespread on the windward island of Hawaii, elevating the number of mosquito species in the island group to 8 [53]. The introduction of mosquitoes and subsequent spread of avian malaria, *Plasmodium relictum*, brought in with exotic birds had disastrous consequences for the native birds of Hawaii, and is considered responsible for the extinction or severe decline of several species [54,55]. In 2003, *Anopheles punctipennis*, a former human malaria vector in North America, was detected on Honolulu but did not establish permanent breeding populations. State quarantine officers in Hawaii have intercepted more than 40 different species of mosquitoes [56]. No terrestrial mammal species occurs in the tropical Pacific islands (including New Caledonia) to the east of the Solomons and west of the Galapagos [57].

### Anophelines and terrestrial mammals

Interestingly, and apparently not previously mentioned in the literature, areas without anopheline species have no native terrestrial mammals. ('Native' and 'terrestrial' are here defined as 'not artificially introduced' and 'non-flying or sea-dwelling', respectively). Anophelines prefer mammals when they are searching for blood meals and indeed, they feed almost entirely on mammals, with an extensive feeding range among mammals depending on their respective availability [58]. Consequently, only a small percentage, less than 1%, of vertebrate blood meals by many *Anopheles *species are from birds or other non-mammalian vertebrates [59]. Observations from our late 2008 fieldwork are in agreement with this general feature, and there appears to be an obligatory relationship for anophelines to feed on terrestrial mammals. Hence, when terrestrial mammals are lacking, anophelines have apparently some behavioural, physiological or metabolic constraints in the use of blood from other terrestrial vertebrates (birds and reptiles) in their reproductive cycle.

As far as can be determined, it is notably rare for bats to be the main source of blood for some very peculiar anophelines; the species *Anopheles hamoni *that lives at all stages in the deepest part of a small number of caves in the Congo feeds mostly on microchiroptera bats [60].

## Conclusions

Any attempt to explain the absence of anophelines in the Seychelles archipelago is inherently speculative. However, the resolution of this question is an interesting and important biogeographic challenge linked with a number of theoretical and applied biological aspects associated with invasive species, specifically the debate of adaptation *vs. *evolution and the aspect of elimination-eradication of natural populations by man.

Biological characteristics of *Anopheles *vectors of human malaria, such as the eggs being susceptible to desiccation, lack of autogeny, preference for natural breeding sites and absence of diapauses, make them relatively inefficient colonizers. However, successful introductions of *An. gambiae s.l. *occurred in the southwestern islands of the Seychelles during the 20^th ^century. Taking into account the importance of the maritime and aerial traffic through the international shipping port and the airport in Victoria, it is highly likely that anophelines have been introduced to the granitic islands, but were unable to establish viable breeding populations. The case of the Seychelles confirms that although they are frequently introduced into non-native areas, and contrarily to mosquitoes belonging to the genera *Aedes *and *Culex*, most introduced anophelines fail to establish permanently. Furthermore, it needs to be underlined that the granitic portions of the Seychelles are also protected by their isolation, remoteness and by head winds mainly blowing from the southeast during a good portion of the year.

Anophelines are well known to feed almost entirely on mammals. The absence of native terrestrial mammals in the few areas of the world that do not harbour anophelines strongly suggests a strict relationship. Apparently bats, even when present, such as on Aldabra, cannot constitute a replacement host to feed on when terrestrial mammals are not available.

## Consent

Written informed consent for publication was obtained from all individuals within the accompanying images.

## Competing interests

The authors declare that they have no competing interests.

## Authors' contributions

VR, GR, SM and SMG conceived the study, made the field survey and wrote the paper. All authors read and approved the final manuscript.
